# The Impact of Mutation of Myelodysplasia-Related Genes in De Novo Acute Myeloid Leukemia Carrying *NPM1* Mutation †

**DOI:** 10.3390/cancers15010198

**Published:** 2022-12-29

**Authors:** Yi Wang, Andres E. Quesada, Zhuang Zuo, L. Jeffrey Medeiros, C. Cameron Yin, Shaoying Li, Jie Xu, Gautam Borthakur, Yisheng Li, Chao Yang, Yasmin Abaza, Juehua Gao, Xinyan Lu, M. James You, Yizhuo Zhang, Pei Lin

**Affiliations:** 1 Department of Hematology, Tianjin Medical University Cancer Institute and Hospital, National Clinical Research Center for Cancer, Key Laboratory of Cancer Prevention and Therapy, Tianjin’s Clinical Research Center for Cancer, Tianjin 300060, China; 2 Department of Hematopathology, The University of Texas MD Anderson Cancer Center, Houston, TX 77030, USA; 3 Department of Leukemia, The University of Texas MD Anderson Cancer Center, Houston, TX 77030, USA; 4 Department of Biostatistics, The University of Texas MD Anderson Cancer Center, Houston, TX 77030, USA; 5 Division of Hematology and Oncology, Department of Medicine, Northwestern University Feinberg School of Medicine, Chicago, IL 60611, USA; 6 Department of Pathology, Northwestern University Feinberg School of Medicine, Chicago, IL 60611, USA; 7 State Key Laboratory of Oncology in South China, Collaborative Innovation Center for Cancer Medicine, Sun Yat-sen University Cancer Center, Guangzhou 510060, China; 8 Department of Pediatric Oncology, Sun Yat-sen University Cancer Center, Guangzhou 510060, China

**Keywords:** AML, *NPM1*, MMD

## Abstract

**Simple Summary:**

*NPM1*-mutated acute myeloid leukemia (AML) is one of the most common subtypes of AML in patients with a normal karyotype. In the recently introduced International Consensus Classification, detection of gene mutations typically associated with myelodysplastic syndrome (MDS) is considered an adverse biomarker in AML patients. However, the impact of these gene mutations occurring in the setting of AML with *NPM1* mutation and without *FLT3*-ITD mutation, a favorable subtype, is unclear. Furthermore, correlation between minimal measurable disease (MMD) with survival in the context of these co-mutations also remains unclear. This study aims to address these issues. We found that patients with or without MDS-related gene mutations treated intensively had a similar MMD rate; however, the former group had a higher relapse rate and shorter progression-free survival (PFS).

**Abstract:**

Background: The impact of gene mutations typically associated with myelodysplastic syndrome (MDS) in acute myeloid leukemia (AML) with *NPM1* mutation is unclear. Methods: Using a cohort of 107 patients with *NPM1*-mutated AML treated with risk-adapted therapy, we compared survival outcomes of patients without MDS-related gene mutations (group A) with those carrying concurrent *FLT3*-ITD (group B) or with MDS-related gene mutations (group C). Minimal measurable disease (MMD) status assessed by multiparameter flow cytometry (MFC), polymerase chain reaction (PCR), and/or next-generation sequencing (NGS) were reviewed. Results: Among the 69 patients treated intensively, group C showed significantly inferior progression-free survival (PFS, *p* < 0.0001) but not overall survival (OS, *p* = 0.055) compared to group A. Though groups A and C had a similar MMD rate, group C patients had a higher relapse rate (*p* = 0.016). Relapse correlated with MMD status at the end of cycle 2 induction (*p =* 0.023). Survival of group C patients was similar to that of group B. Conclusion: MDS-related gene mutations are associated with an inferior survival in *NPM1*-mutated AML.

## 1. Introduction

Acute myeloid leukemia (AML) with *NPM1* mutation and a normal karyotype comprises 30% of AML and is one of the most common subtypes [1,2,3]. This group is clinically heterogeneous with variable responses to therapy and outcomes likely attributable, at least in part, to coexisting mutations of other genes. While cases with concurrent *FLT3*-ITD^high^ (allelic ratio (AR) > 0.5) were previously designated as “intermediate-risk” based on the European LeukemiaNet (ELN) risk stratification [4,5], the newly updated ELN classification now categorizes all cases with *NPM1*-mutated AML with *FLT3*-ITD co-mutation as intermediate-risk group regardless of AR [6].

Historically, patients who developed AML preceded by a history of myelodysplastic syndrome (MDS) or with cytogenetic aberrations associated with MDS (-7/7q-, complex, etc.) were designated as AML with myelodysplasia-related changes (AML-MRC) rather than AML with *NPM1* mutation even if the mutation was detected [7]. The recently introduced International Consensus Classifications (ICC) and ELN risk stratification proposed to include cases with gene mutations typically associated with MDS as the third pathway to the category of AML-MRC, designated as AML with myelodysplasia-related gene mutations [6,8]. A pathological variant of any of *ASXL1, BCOR, EZH2, RUNX1, SF3B1, SRSF2, STAG2, U2AF1*, or *ZRSR2* genes denotes MDS-related gene mutations. Studies have shown their prevalence in secondary AML [9]. However, both ICC and ELN acknowledged that the impact of these mutations in *NPM1*-mutated AML is less clear. Additionally, AML with *TP53* mutation and *WT1* mutation has been shown to be aggressive [3], and yet, its role in *NPM1*-mutated AML is also unclear. To address this issue, we designed and performed the current study comparing survival of three subgroups of *NPM1* mutated AML: group A, without MDS-related gene mutations; group B, with *FLT3*-ITD mutation regardless of AR; and group C, with MDS-related gene mutations. For this study, *TP53*-mutated cases were included in group C, as it is also commonly seen in secondary AML [3,9,10,11]. Cases with co-mutation of *WT1* but without *FLT3*-ITD were excluded from group C and designated as group D.

Pretreatment variables such as age, performance status, white blood cell (WBC) count, and molecular genetic data currently represent the best-established, albeit imperfect, predictors of response to therapy [12]. Increasingly, post-treatment factors such as MMD have been evaluated as a surrogate of treatment efficacy [13]. MMD can be assessed by multi-color flow cytometry (MFC) or molecular genetic methods such as reverse transcriptase polymerase chain reaction (RT-PCR) methods or next-generation sequencing (NGS) [14]. The relationship between mutation profile, MMD status, and outcome are not fully described in AML with mutations of *NPM1*- and MDS-related genes.

In the current study, we evaluated 107 consecutive patients of de novo AML with *NPM1* mutation. MMD status assessed by various combinations of MFC, PCR, and NGS was correlated with mutation profiles as well as survival.

## 2. Materials and Methods

### 2.1. Patients

We searched databases in MD Anderson Cancer Center and Northwestern University Feinberg School of Medicine from 1 January 2012 through 31 December 2018 for patients diagnosed with de novo AML associated with mutated *NPM1*. AML with other recurrent genetic aberrations or secondary/transformed AML was excluded [15]. All patients had a normal karyotype except for 5 patients who had abnormalities commonly associated with *NPM1* mutation. These abnormalities have not been shown to affect prognosis [5,7,16,17], including one patient with –Y, one with del(9q), one with +8, one with -16q, and one patient with +8, +8, and dup(13) [18,19]. Clinical and laboratory data were obtained by review of the medical records in accordance with the institutional internal review-board-approved protocols.

### 2.2. Treatment and Follow-Up

Patients were treated based on clinical assessment and institutional protocols. Patients who were treated intensively received either Fludarabine + Ara-C + G-CSF + Idarubicin (FLAG-IDA), Cladribine combined with Idarubicin and Ara-C (CLIA) with or without venetoclax, or standard “3 + 7”. Alternatively, non-intensive regimens included hypomethylating agents (HMA) with or without venetoclax or Cladribine or low-dose Ara-C alternating with decitabine. FLT3 or IDH inhibitors were added in the appropriate context. All patients with *FLT3*-ITD mutation were treated with FLT3 inhibitor. A subset of intensively treated patients received allogeneic hematopoietic stem cell transplantation (HSCT).

### 2.3. Multiparameter Flow Cytometric (MFC) Immunophenotyping

Initial and post-treatment bone marrow aspirate samples were processed using standard methods and analyzed using FACS Canto II 8-color instruments (BD Biosciences, San Diego, CA, USA). Details of the assay have been previously described [20,21]. The detection sensitivity is generally 0.1–1% in an adequate sample.

### 2.4. Polymerase Chain Reaction (PCR) and Next-Generation Sequencing (NGS)

*FLT3*-ITD and tyrosine kinase domain (TKD) mutations and *NPM1* mutations were identified using PCR-based methods followed by capillary electrophoresis on Genetic Analyzer (Applied Biosystems, Foster City, CA, USA), as described previously [22,23]. The sensitivity level of these PCR assays is 1% and can detect most common (A, B, and D) types of *NPM1* mutations. High-throughput sequencing was performed using a MiSeq sequencer (Illumina Inc., San Diego, CA, USA), and data were analyzed using MiSeq Reporter Software. A 28-gene NGS-panel assessed full exons for mutations in the following genes: *ABL, EGFR, GATA2, IKZF2, MDM2, NOTCH1, RUNX1, ASXL1, EZH2, HRAS, JAK2, MLL, NPM1, TET2, BRAF, FLT3, IDH1, KIT, MPL, NRAS, TP53, DNMT3A, GATA1, IDH2, KRAS, MYD88, PTPN11,* and *WT1*. In a subset of cases, an 81-gene panel was also performed (Table S1). Adequate coverage was defined as ≥ 250 reads for each exon. The analytical sensitivity of the platform is variable for different genes but is generally 2.5% for most genes; the sensitivity of the *NPM1* p.W288 locus with manual review of IGV reads is 0.01–0.1% [21].

### 2.5. Mutational Profile and Assignment of Molecular Risk Groups

NGS analysis was performed on the initial diagnostic and post-treatment samples. Results of *FLT3*-ITD and *NPM1* analyzed by PCR were used in conjunction with NGS to stratify patients into four risk groups using published models as guidelines (Table S2) [2,11,24]. Specifically, group A consisted of patients without mutations of *ASXL1, BCOR, EZH2, RUNX1, SF3B1, SRSF2, STAG2, U2AF1, ZRSR2, MLL*, *TP53, WT1,* or *FLT3*-ITD [25]; group B: concurrent *FLT3*-ITD regardless of AR level; and group C: concurrent mutations of any of the MDS-related genes listed above. Cases with *WT1* mutation but without *FLT3*-ITD were designated as group D [26,27,28]. Due to the limited size of group D (N = 4), no further survival comparison was performed.

### 2.6. Statistical Analysis

Survival distributions were estimated by the Kaplan–Meier method (Log-rank test) using Graph-Pad software (Prism, San Diego, CA, USA); unpaired *t*-test, Fisher’s exact test, and chi-square test were performed to assess the clinical characteristics and treatment response of each molecular risk group. Cox proportional hazards models were estimated with regards to various parameters by R software version 4.0.3 (Vienna, Austria).

Complete remission (CR) and CR with incomplete blood count recovery (CRi) are defined according to the standard criteria [29,30]. For the purpose of this study, we classified those that failed to achieve CR by the end of first cycle of induction (EOC1) (day 28–35) as delayed CR. The best response achieved was noted for each patient. MMD status was determined once the patients met the criteria for morphologically free of leukemia state (MLFS) (<5% blasts by morphology) or CR/CRi, which was usually assessed at EOC1 and EOC2. *DNMT3A, IDH1/2, ASXL1, TET2,* or *SRSF2* were considered as underlying clonal hematopoiesis of undetermined significance (CHIP). Survival was determined from the date of diagnosis to date of death from any cause or last follow-up.

Statistical analyses were performed by two biostatisticians independently.

## 3. Results

**Figure 1:**
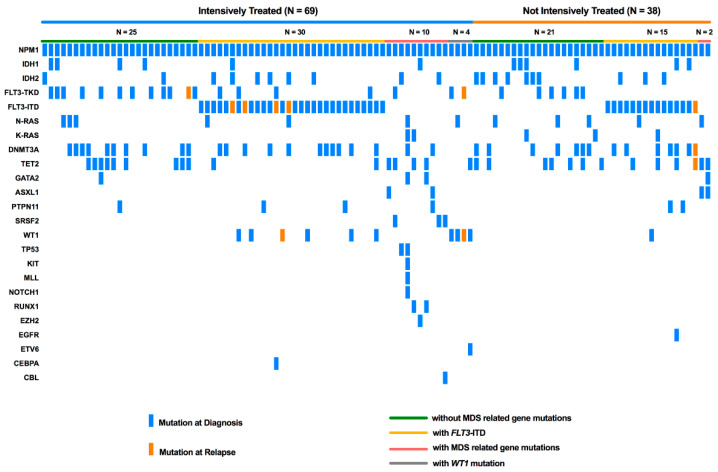
Mutational landscape of 107 cases of *NPM1* mutated AML. Mutations of genes acquired at relapse are highlighted in orange and those at diagnosis in blue. Each column represents a patient.

The mutation profiles of all 107 cases are summarized in Table S2 and illustrated in Figure 1. Sixty-nine patients were treated with intensive chemotherapy, whereas thirty-eight patients were treated with less-intensive regimens. The demographic and hematologic features of the two groups are summarized in Table 1. The median follow-up for the entire cohort was 30.1 months (range, 2.1–84.8 months). 

Compared with the intensively treated group, the not-intensively treated group was older (median age 72, range 23–87 years) (*p* < 0.0001) and MMD was more frequently detected in 26/34 (76.47%) patients (*p* < 0.0001) (Table 1). At last follow-up, 31 (81.58%) patients had died, and the median OS was 18.1 months (range, 2.8–79.4 months). In contrast, the median age of the intensively treated group of patients was 53 years (range, 17–69 years). This group was then the main focus of MMD study and survival comparison among the three subgroups are detailed in Table 2.

### 3.1. Correlation between MMD and Relapse and Survival

**Figure 2:**
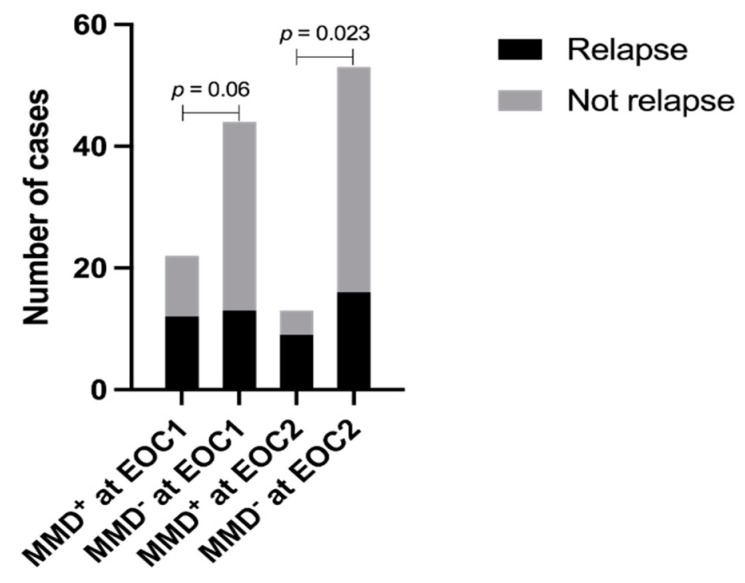
Correlation of MMD status at EOC1 and EOC2 with relapse. MMD, minimal measurable disease; EOC1, end of cycle 1 induction; EOC2, end of cycle 2 induction.

We identified MMD in 22 (22/66, 33.33%) and 13 (13/66, 19.70%) patients at EOC1 and EOC2, respectively, seen across all the three subgroups (A, B, C) at a similar rate (Table 2). However, group B and C patients had a higher relapse rate (*p* = 0.0009) (Table 2). Among the 27 patients who eventually had relapsed AML, 12 (12/25, 48%) patients had detectable MMD at EOC1, and 9 (9/25, 36.00%) had MMD at EOC2. Only MMD at EOC2 correlated with relapse (*p* = 0.023) (Figure 2).

**Figure 3:**
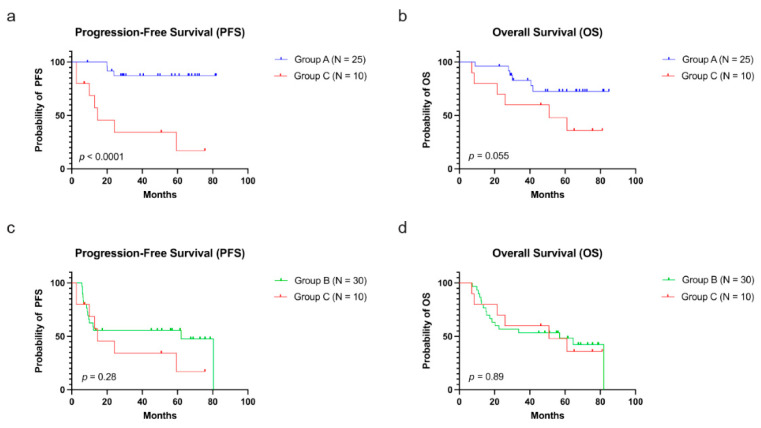
Kaplan–Meier curves comparing groups A and C show significant difference in PFS (**a**) but not OS (**b**). Kaplan–Meier curves comparing groups B and C show no significant difference in PFS (**c**) or OS (**d**). Group A, without MDS-related gene mutations; Group B, with *FLT3*-ITD; Group C, with MDS-related gene mutations.

Kaplan–Meier survival analysis showed a significant survival difference among group A, B, and C (Table 2). The median OS and PFS of the patients in group A were not yet reached. The median OS and PFS of the patients in group B are 56.90 and 62.10 months, respectively. The median OS and PFS of the patients in group C are 50.80 and 14.60 months, respectively (Table 2). Consistent with previous studies, the subgroup with *FLT3*-ITD showed significantly higher relapse rate and worse survival than patients of group A (Table S3). Most notably, group C showed worse PFS (Figure 3a, *p* < 0.0001) but not OS (Figure 3b, *p* = 0.055) than group A (Table 3). Comparison of patients in group B and C found no significant differences in relapse rate (*p* = 0.46), PFS (Figure 3c, *p* = 0.28), or OS (Figure 3d, *p* = 0.89) (Table S4). In addition, among the 69 patients treated intensively, no PFS or OS differences were noted among patients who were treated with HSCT (N = 43) vs. those without (N = 26) (*p =* 0.95 and *p =* 0.32, respectively). Survival curves were censored for patients who received HSCT from the date of transplantation (Table 2).

At relapse, all patients retained *NPM1* mutation, and eight patients acquired additional mutations not seen at initial diagnosis, including *FLT3*-ITD in five, *FLT3*-TKD in two, and *WT1* in two. Additionally, six patients who initially had a normal karyotype developed an abnormal karyotype, and 5/6 died within 12 months (Table S5). Multivariate Cox proportional hazard analysis showed that both *FLT3*-ITD- and MDS-related gene mutations were independent prognostic markers (Table 4).

### 3.2. Comparison of MFC to Molecular Testing

A total of 151, 459, and 559 post-therapy samples were analyzed by NGS, MFC, and PCR, respectively. Discordant results between MFC and molecular testing were observed in 30 samples, and the discordance was predominantly due to a negative or indeterminate MFC result compared with a positive molecular result; in particular, *NPM1* or *FLT3*-ITD were most commonly detected by PCR or rarely by NGS.

## 4. Discussion

The ELN risk stratification for *NPM1*-mutated AML based solely on *FLT3*-ITD is now presumed to represent an oversimplified model for risk assessment [5,7,13,28,31]. In recent years, NGS studies have refined risk stratification among patients with intermediate-risk AML [3,28,32]. Most recently, the ICC has introduced a new pathway to arrive at the diagnosis of AML-MRC based on mutation of genes typically associated with MDS, designated as AML with MDS-related gene mutations. The new category involves pathological variants of *ASXL1*, *BCOR*, *EZH2*, *RUNX1*, *SF3B1*, *SRSF2*, *STAG2*, *U2AF1,* and *ZRSR2*. AML with *TP53* mutation is also a new category in the ICC given its adverse effect on many subtypes of AML and its common occurrence in secondary AML. An AML with *NPM1* mutation and without *FLT3*-ITD is considered a favorable subtype; however, the impact of MDS-related gene mutations on the specific subtype is unclear or controversial [32]. In addition, the new classification recognizes all *FLT3*-ITD, regardless of AR, as an adverse risk factor.

To further explore the issue, we stratified patients with *NPM1*-mutated AML as unfavorable risk, along with *FLT3*-ITD, based on presence of these MDS-associated gene mutations and *TP53* mutations using NGS profiling. *WT1*-mutated AML has been shown to adversely impact survival and is proposed as a distinct molecular subgroup of AML [3,11,26,27,28]. Thus, we designated *WT1*-mutated cases as group D, separate from the other subgroups.

Patients in the group C treated with intensive regimens showed a similar rate of treatment response compared to group A. The MMD rate was also similar at ECO1 and ECO2. However, group C patients suffered a higher rate of relapse as well as shorter PFS compared to group A. The PFS and OS of the group C patients were comparable to those of group B. These findings support incorporating these secondary (MDS)-related mutation profiles into the risk stratification.

Detectable MMD at EOC2 but not EOC1 correlated with relapse. This finding supports the notion that delayed blast clearance is an adverse risk factor [33] and the consensus recommendation that MMD testing is most informative at EOC2 [14]. For patients who had relapse, all had molecular evidence of disease. Because our PCR and NGS assays for *NPM1* have a sensitivity level of 0.01–1%, it was significant whenever abnormal variants were detected. However, samples obtained at EOC1 often and sometimes EOC2 are hypocellular, potentially limiting MFC assay sensitivity. Thus, not unexpectedly, we observed discordant results between MFC and molecular methods.

*DNMT3A, ASXL1, TET2, IDH1/2,* and *SRSF2* are now recognized as CHIP-like mutations that can persist during CR [34,35]. In contrast to CHIP, clonal hematopoiesis of oncogenic potential (CHOP)-type mutations that persist after therapy are more ominous [36,37]. The high frequency of relapse in group C patients suggests that the MDS-related gene mutations, like CHIP, may predispose the patients to secondary/relapsed AML. In fact, concurrent *DNMT3A* mutation was shown to have no adverse impact on survival but more likely correlated with MMD positivity and potentially increased the risk for relapse or secondary AML [2]. Despite a similar rate of MMD compared to group A patients, group C patients had a higher relapse rate, suggesting that the MDS-related gene mutations are implicated in the inferior survival.

## 5. Conclusions

The mutation profiles of *NPM1*-mutated AML have implications in patient survival in the current era of risk-adapted therapy. In addition to *FLT3*-ITD, concurrent MDS-related gene mutations are associated with an inferior outcome, supporting their recognition in *NPM*1-mutated AML. The mechanisms of this phenomenon remain to be explored, but they are most likely related to persistence of underlying preleukemic clone and unremitted risk for genetic alterations in affected patients despite achieving CR.

## Figures and Tables

**Table 1:** Summary of clinicopathological features of the total study cohort.

	Intensively Treated	Not Intensively Treated	*p*-Value
Number	69	38	N/A
Age (years) median and range	53, (17–69)	72, (23–87)	**<0.0001**
Sex:			
-Men -Women	35 (50.72%) 34 (49.28%)	18 (47.37%) 20 (52.63%)	0.84
BM blasts (%) median and range	64, (1–95)	59.5, (6–90)	0.96
WBC (k/µL) median and range	12.25, (0.1–378.4)	18.55, (0.9–140)	0.70
Response:			
-CR/CRi	56/68 (82.35%)	33/37 (89.19%)	0.06
-MLFS	10/68 (14.71%)	1/37 (2.70%)	
-Refractory	2/68 (2.94%)	3/37 (8.11%)	
MMD (EOC1)	22/66 (33.33%)	26/34 (76.47%)	**<0.0001**
Relapse	27 (39.13%)	20 (52.63%)	0.22
HSCT	43 (62.32%)	3 (7.89%)	**<0.0001**
Mutation profiles:			
-Group A	25 (36.23%)	21 (55.26%)	
-Group B	30 (43.48%)	15 (39.47%)	
-Group C	10 (14.49%)	2 (5.26%)	
*TP53*	2	0	
*RUNX1*/*ASXL1*/*SRSF2*/*EZH2*	2/2/3/1	0/2/0/0	
-Group D	4 (5.80%)	0 (0.00%)	

BM, bone marrow; WBC, white blood cell; CR, complete remission; CRi, CR with incomplete blood count recovery; MLFS, morphologic leukemia-free state; MMD, minimal measurable disease; EOC1, end of cycle 1 induction; HSCT, hematopoietic stem cell transplantation; Group A, without MDS-related gene mutations; Group B, with *FLT3*-ITD; Group C, with MDS-related gene mutations; Group D, with *WT1* mutation. *p*-values in bold are of statistical significance.

**Table 2:** Comparison between different risk groups of patients treated with intensive chemotherapy.

	Group A	Group B	Group C	*p*-Value
Number	25	30	10	N/A
Age (years) median and range	57, (17–69)	51.5, (19–67)	59, (33–68)	0.58
Age > 65 years	4 (16.00%)	5 (16.67%)	3 (30%)	0.59
Sex:				
-Men -Women	13 (52.00%) 12 (48.00%)	14 (46.67%) 16 (53.33%)	6 (60.00%) 4 (40.00%)	0.76
BM blasts (%) median and range	51.5, (4–94)	68, (1–95)	67, (19–86)	0.48
WBC (k/µL) median and range	11.2, (0.5–378.4)	14.9, (1–160.4)	17.95, (1–44.1)	0.37
PB blasts (%) median and range	23.5, (0–97)	35.5, (0–98)	16, (0–83)	0.43
Hb (g/dL) median and range	8.95, (5.1–12.4)	9.35, (7.7–15.5)	9.15, (8.5–14.4)	0.17
platelets (k/µL) median and range	53, (11–385)	57.5, (9–553)	29.5, (7–87)	0.18
Response:				
-CR/CRi	23/25 (92.00%)	22/29 (75.86%)	7/10 (70.00%)	**0.028**
-MLFS	2/25 (8.00%)	7/29 (24.14%)	1/10 (10.00%)	
-Refractory	0 (0.00%)	0 (0.00%)	2/10 (20.00%)	
MMD (EOC1)	6/25 (24.00%)	11/29 (37.93%)	5/8 (62.50%)	0.13
MMD (EOC2)	4/25 (16.00%)	7/29 (24.14%)	2/8 (25.00%)	0.68
Relapse	3 (12.00%)	15 (50.00%)	7 (70.00%)	**0.0009**
Median OS (months)	NR	56.90	50.80	**0.045**
Median OS (months) (HSCT censored)	NR	NR	NR	0.14
Median PFS (months)	NR	62.10	14.60	**0.0006**
Median PFS (months) (HSCT censored)	NR	NR	24.3	**0.0014**

All values taken at diagnosis; normal range, WBC, 4.0–11.0 k/µL; hemoglobin, 14–18 g/dL; platelets, 140–440 k/µL; Group A, without MDS-related gene mutations; Group B, with *FLT3*-ITD; Group C, with MDS-related gene mutations; BM, bone marrow; WBC, white blood cell; PB, peripheral blood; Hb, hemoglobin; CR, complete remission; CRi, CR with incomplete blood count recovery; MLFS, morphologic leukemia-free state; MMD, minimal measurable disease; EOC1, end of cycle 1 induction; EOC2, end of cycle 2 induction; OS, overall survival; HSCT, hematopoietic stem cell transplantation; PFS, progression-free survival; NR, median survival not reached. *p*-values in bold are of statistical significance.

**Table 3:** Comparison between patients without (Group A) versus with (Group C) MDS-related gene mutations treated with intensive chemotherapy.

	Group A	Group C	*p*-Value
Number	25	10	N/A
Age (years) median and range	57, (17–69)	59, (33–68)	0.90
Age > 65 years	4 (16.00%)	3 (30%)	0.38
Sex:			
-Men -Women	13 (52.00%) 12 (48.00%)	6 (60.00%) 4 (40.00%)	0.72
BM blasts (%) median and range	51.5, (4–94)	67, (19–86)	0.87
WBC (k/µL) median and range	11.2, (0.5–378.4)	17.95, (1–44.1)	0.25
PB blasts (%) median and range	23.5, (0–97)	16, (0–83)	0.42
Hb (g/dL) median and range	8.95, (5.1–12.4)	9.15, (8.5–14.4)	0.09
platelets (k/µL) median and range	53, (11–385)	29.5, (7–87)	0.13
Response:			
-CR/CRi	23/25 (92.00%)	7/10 (70.00%)	0.09
-MLFS	2/25 (8.00%)	1/10 (10.00%)	
-Refractory	0 (0.00%)	2/10 (20.00%)	
MMD (EOC1)	6/25 (24.00%)	5/8 (62.50%)	0.08
MMD (EOC2)	4/25 (16.00%)	2/8 (25.00%)	0.62
Relapse	3 (12.00%)	7 (70.00%)	**0.0016**
Median OS (months)	NR	50.80	0.055
Median OS (months) (HSCT censored)	NR	NR	0.051
Median PFS (months)	NR	14.60	**<0.0001**
Median PFS (months) (HSCT censored)	NR	24.3	**<0.0001**

All values taken at diagnosis; normal range, WBC, 4.0–11.0 k/µL; hemoglobin, 14–18 g/dL; platelets, 140–440 k/µL; Group A, without MDS-related gene mutations; Group C, with MDS-related gene mutations; BM, bone marrow; WBC, white blood cell; PB, peripheral blood; Hb, hemoglobin; CR, complete remission; CRi, CR with incomplete blood count recovery; MLFS, morphologic leukemia-free state; MMD, minimal measurable disease; EOC1, end of cycle 1 induction; EOC2, end of cycle 2 induction; OS, overall survival; HSCT, hematopoietic stem cell transplantation; PFS, progression-free survival; NR, median survival not reached. *p*-values in bold are of statistical significance.

**Table 4:** Multivariate Cox proportional hazards model for overall survival (OS) and progression free survival (PFS) in patients treated with intensive chemotherapy.

Variable	Hazard Ratio	95% CI	*p*-Value
**Overall Survival (OS)**			
-WBC count (>100 k/µL)	3.73	(1.14, 12.14)	**0.029**
-BM blast (%)	0.98	(0.97, 1.00)	0.11
-Age (>65 years)	0.83	(0.22, 3.20)	0.79
-MMD (EOC2)	1.49	(0.58, 3.84)	0.41
-Group (A as reference)			
B	2.77	(1.05, 7.32)	**0.040**
C	3.18	(0.86, 11.80)	0.08
**Progression-Free Survival (PFS)**			
-WBC count (>100 k/µL)	0.93	(0.23, 3.73)	0.91
-BM blast (%)	1.01	(0.99, 1.03)	0.31
-Age (>65 years)	1.78	(0.51, 6.25)	0.37
-MMD (EOC2)	2.34	(0.88, 6.23)	0.09
-Group (A as reference)			
B	5.16	(1.42, 18.69)	**0.013**
C	7.65	(1.70, 34.48)	**0.0080**

WBC stratified as below: <50, 50–100, >100 K; WBC, white blood cell; BM, bone marrow; MMD, minimal measurable disease; EOC2, end of cycle 2 induction; Group A, without MDS-related gene mutations; Group B, with *FLT3*-ITD; Group C, with MDS-related gene mutations. *p*-values in bold are of statistical significance.

## Data Availability

Not applicable.

## Supplementary Materials

The following supporting information can be downloaded at: https://www.mdpi.com/article/10.3390/cancers15010198/s1, Table S1: Eighty-one gene NGS panel; Table S2: Mutational Profile and Risk Group Assignment; Table S3: Comparison between subgroups of patients without MDS-related gene mutations (Group A) versus with *FLT3*-ITD (Group B) treated with intensive chemotherapy. Table S4: Comparison between subgroups of patients with *FLT3*-ITD (Group B) versus with MDS-related gene mutations (Group C) treated with intensive chemotherapy; Table S5: Abnormal karyotype identified at or after relapse.
