# Supplementary material for: The Impact of Mutation of Myelodysplasia-Related Genes in De Novo Acute Myeloid Leukemia Carrying NPM1 Mutation
Source: Cancers (Basel). 2022 Dec 29;15(1):198. doi: 10.3390/cancers15010198 (PMC9818485; doi:10.3390/cancers15010198)
Supplement: Supplementary file 1 [file cancers-15-00198-s001.zip › cancers-1979691-supplementary.pdf]

Supplementary Materials

Table S1. Eighty-one-gene NGS panel.

ANKRD26	*GNAS*	*KRAS*	*RAD21*	*STAT5A*
ASXL1	*HNRNPK*	*MAP2K1*	*RARA*	*STAT5B*
ASXL2	*HRAS*	*MPL*	*RUNX1*	*SUZ12*
BCOR	*IDH1*	*NF1*	*SETBP1*	*TERC*
BCORL1	*IDH2*	*NOTCH1*	*SF1*	*TERT*
BRAF	*IKZF1*	*NPM1*	*SF3A1*	*TET2*
BRINP3	*IL2RG*	*NRAS*	*SF3B1*	*TP53*
CALR	*IL7R*	*PAX5*	*SH2B3*	*U2AF1*
CBL	*JAK1*	*PHF6*	*SMC1A*	*U2AF2*
CBLB	*JAK2*	*PIGA*	*SMC3*	*WT1*
CBLC	*JAK3*	*PML*	*SRSF2*	*ZRSR2*
CEBPA	*KDM6A*	*PRPF40B*	*STAG1*	
CREBBP	*KIT*	*PTEN*	*STAG2*	
CRLF2	*KMT2A*	*PTPN11*	*STAT3*	

Table S2. Mutational Profile and Risk Group Assignment.

Case Number	Risk Group	Mutational Profile
1	A	*NPM1, IDH2,*
2	A	*NPN1, IDH1, FLT3*-D835
3	A	*NPM1, IDH1, FLT3*-D835
4	A	*NPM1, FLT3*-D835, *N-RAS*
5	A	*NPM1, DNMT3A, N-RAS*
6	A	*NPM1, DNMT3A, N-RAS,*
7	A	*NPM1, DNMT3A, FLT3*-TKD
8	B	*NPM1, FLT3*-ITD
9	B	*NPM1, FLT3*-ITD, *N-RAS*
10	B	*NPM1, FLT3*-ITD, *TET2, IDH2*
11	B	*NPM1, FLT3*-ITD, *FLT3*-D835, *DNMT3A*
12	A	*NPM1, TET2, DNMT3A*
13	A	*NPM1, TET2*
14	A	*NPM1, TET2, FLT3*-D835, *GATA2*
15	A	*NPM1, TET2, DNMT3A*
16	A	*NPM1, DNMT3A, TET2*
17	B	*NPM1, FLT3*-ITD
18	C	*NPM1, ASXL1, TET2, TET2*
19	B	*NPM1, DNMT3A, FLT3*-ITD
20	A	*NPM1, PTPN11, IDH1, FLT3*-D835
21	A	*NPM1, DNMT3A, TET2*
22	A	*NPM1, FLT*-D835
23	A	*NPM1*
24	A	*NPM1, DNMT3A, IDH1*
25	A	*NPM1, FLT3*-D835
26	A	*NPM1*
27	C	*NPM1, TET2, FLT3*-D835, *SRSF2*
28	A	*NPM1, IDH2, FLT3*-D835
29	B	*NPM1, IDH1, IDH2, FLT3*-ITD
30	B	*NPM1, NPM1, FLT3*-ITD, *WT1*
31	B	*NPM1, FLT3*-ITD, *WT1*

32	B	*NPM1*, *DNMT3A*, *FLT3*-ITD
33	D	*NPM1*, *FLT3*-D835, *WT1*
34	B	*NPM1*, *FLT3*-ITD, *IDH2*
35	B	*NPM1*, *FLT3*-ITD, *WT1*
36	B	*NPM1*, *FLT3*-ITD, *DNMT3A*
37	B	*NPM1*, *FLT3*-ITD, *DNMT3A*
38	B	*NPM1*, *FLT3*-ITD, *DNMT3A*
39	B	*NPM1*, *FLT3*-ITD, *DNMT3A*, *NOTCH1*
40	B	*NPM1*, *FLT3*-ITD, *PTPN11*
41	C	*NPM1*, *IDH2*, *TP53*
42	C	*NPM1*, *TP53*, *DNMT3A*, *N-RAS*, *K-RAS*, *KIT*, *MLL*, *GATA2*, *NOTCH1*
43	B	*NPM1*, *IDH2*, *FLT3*-ITD
44	B	*NPM1*, *DNMT3A*, *FLT3*-ITD, *WT1*
45	A	*NPM1*, *FLT3*-TKD
46	B	*NPM1*, *PTPN11*, *FLT3*-ITD
47	B	*NPM1*, *FLT3*-ITD, *FLT3*-ITD, *FLT3*-ITD
48	A	*NPM1*, *TET2*, *TET2*
49	D	*NPM1*, *N-RAS*, *WT1*
50	B	*NPM1*, *FLT3*-ITD
51	B	*NPM1*, *DNMT3A*, *IDH2*, *FLT3*-ITD
52	B	*NPM1*, *FLT3*-ITD, *FLT3*-ITD, *FLT3*-ITD, *FLT3*-D835†
53	A	*NPM1*, *DNMT3A*. *TET2*, *TET2*
54	A	*NPM1*, *TET2*, *DNMT3A*, *FLT3*-V592G
55	B	*NPM1*, *FLT3*-ITD, *FLT3*-TKD, *FLT3*-ITD, *CEBPA*
56	C	*NPM1*, *K-RAS*, *RUNX1*, *TET2*, *TET2*, *TET2*
57	C	*NPM1*, *EZH2*, *IDH1*
58	B	*NPM1*, *DNMT3A*, *FLT3*-ITD, *TET2*, *WT1*†
59	C	*NPM1*, *TET2*, *GATA2*, *RUNX1*
60	B	*NPM1*, *FLT3*-ITD
61	D	*NPM1*, *FLT3*-D835, *WT1*†
62	C	*NPM1*, *DNMT3A*, *PTPN11*, *ASXL1*
63	B	*NPM1*, *FLT3*-ITD, *WT1*, *WT1*†
64	B	*NPM1*, *DNMT3A*, *IDH2*, *N-RAS*, *FLT3*-ITD
65	B	*NPM1*, *FLT3*-ITD, †
66	D	*NPM1*, *WT1*, *ETV6*, *TET2*
67	A	*NPM1*, *FLT3*-TKD
68	C	*NPM1*, *IDH2*, *SRSF2*
69	C	*NPM1*, *SRSF2*, *CBL*
70	A	*NPM1*, *TET2*, *IDH2*, *DNMT3A*
71	A	*NPM1*, *IDH2*
72	A	*NPM1*, *TET2*, *DNMT3A*
73	B	*NPM1*, *FLT3*-ITD
74	B	*NPM1*, *DNMT3A*, *FLT3*-ITD
75	A	*NPM1*, *IDH2*, *N-RAS*
76	B	*NPM1*, *FLT3*-ITD, *IDH2*, *NOTCH*
77	A	*NPM1*, *FLT3*-D835
78	A	*NPM1*, *IDH2*
79	A	*NPM1*, *IDH1*
80	B	*NPM1*, *DNMT3A*, *FLT3*-ITD
81	A	*NPM1*, *IDH1*

82	A	*NPM1, IDH1, IDH2, K-RAS*
83	A	*NPM1, DNMT3A, IDH2*
84	A	*NPM1, IDH2, FLT3*-V592G
85	A	*NPM1, TET2, TET2*
86	A	*NPM1, FLT3*-TKD, *TET2, TET2*
87	B	*NPM1, TET2, FLT3*-ITD
88	C	*NPM1, ASXL1, N-RAS, TET2*
89	A	*NPM1, DNMT3A, N-RAS*
90	A	*NPM1, FLT3*-TKD
91	C	*NPM1, ASXL1, GATA2, TET2*
92	A	*NPM1, TET2, TET2*
93	A	*NPM1, DNMT3A, IDH1, FLT3*-TKD
94	B	*NPM1, FLT3*-ITD, *N-RAS*
95	A	*NPM1, TET2, FLT3*-TKD, *DNMT3A*
96	A	*NPM1, DNMT3A, N-RAS*
97	A	*NPM1, K-RAS*
98	B	*NPM1, FLT3*-ITD, *IDH2*
99	A	*NPM1, TET2*
100	B	*NPM1, WT1, FLT3*-ITD
101	B	*NPM1, FLT3*-ITD, *TET2, DNMT3A, K-RAS*
102	B	*NPM1, FLT3*-ITD, *IDH2*
103	B	*NPM1, DNMT3A, FLT3*-ITD, *PTPN11*
104	B	*NPM1, DNMT3A, DNMT3A, IDH1, TET2, FLT3*-ITD, *EGFR*
105	B	*NPM1, FLT3*-ITD, *PTPN11*
106	B	*NPM1, DNMT3A, IDH1, FLT3*-ITD
107	B	*NPM1, FLT3*-ITD, *TET2, DNMT3A*

Cases 1–69 were treated intensively, and cases 70–107 were treated with reduced intensity; risk-group assignment: Group A, without MDS-related gene mutations; Group B, with *FLT3*-ITD; Group C, with MDS-related gene mutations; Group D, with *WT1* mutation. Mutations gained at relapse are in red; †, abnormal cytogenetics detected at relapse or shortly after.

Table S3. Comparison between subgroups of patients without MDS-related gene mutations (Group A) versus with *FLT3*-ITD (Group B) treated with intensive chemotherapy.

	Group A	Group B	*p*-Value
Number	25	30	N/A
Age (years) median and range	57, (17–69)	51.5, (19–67)	0.30
Age > 65 years	4 (16.00%)	5 (16.67%)	>0.9999
Sex:			
-Men	13 (52.00%)	14 (46.67%)	0.79
-Women	12 (48.00%)	16 (53.33%)	
BM blasts (%) median and range	51.5, (4–94)	68, (1–95)	0.25
WBC (k/μL) median and range	11.2, (0.5–378.4)	14.9, (1–160.4)	0.46
PB blasts (%)median and range	23.5, (0–97)	35.5, (0–98)	0.58
Hb (g/dL) median and range	8.95, (5.1–12.4)	9.35, (7.7–15.5)	0.16
platelets (k/μL) median and range	53, (11–385)	57.5, (9–553)	0.38
Response:			
-CR/CRi	23/25 (92.00%)	22/29 (75.86%)	0.15
-MLFS	2/25 (8.00%)	7/29 (24.14%)	
-Refractory	0 (0.00%)	0 (0.00%)	
MMD (EOC1)	6/25 (24.00%)	11/29 (37.93%)	0.38
MMD (EOC2)	4/25 (16.00%)	7/29 (24.14%)	0.52
Relapse	3 (12.00%)	15 (50.00%)	**0.0037**

Median OS (months)	NR	56.90	**0.017**
Median OS (months) (HSCT censored)	NR	NR	0.15
Median PFS (months)	NR	62.10	**0.0013**
Median PFS (months) (HSCT censored)	NR	NR	**0.019**

All values taken at diagnosis; normal range, WBC, 4.0–11.0 k/μL; hemoglobin, 14–18 g/dL; platelets, 140–440 k/μL; Group A, without MDS-related gene mutations; Group B, with *FLT3*-ITD; BM, bone marrow; WBC, white blood cell; PB, peripheral blood; Hb, hemoglobin; CR, complete remission; Cri, CR with incomplete blood count recovery; MLFS, morphological leukemia-free status; MMD, minimal measurable disease; EOC1, end of cycle 1 induction; EOC2, end of cycle 2 induction; OS, overall survival; HSCT, hematopoietic stem cell transplantation; PFS, progression-free survival; NR, median survival not reached. *p*-values in bold are of statistical significance.

Table S4. Comparison between subgroups of patients with *FLT3*-ITD (Group B) versus with MDS-related gene mutations (Group C) treated with intensive chemotherapy.

	Group B	Group C	*p*-Value
Number	30	10	N/A
Age (years) median and range	51.5, (19–67)	59, (33–68)	0.55
Age > 65 years	5 (16.67%)	3 (30%)	0.39
Sex:			
-Men	14 (46.67%)	6 (60.00%)	0.72
-Women	16 (53.33%)	4 (40.00%)	
BM blasts (%) median and range	68, (1–95)	67, (19–86)	0.50
WBC (k/μL) median and range	14.9, (1–160.4)	17.95, (1–44.1)	0.22
PB blasts (%)median and range	35.5, (0–98)	16, (0–83)	0.18
Hb (g/dL) median and range	9.35, (7.7–15.5)	9.15, (8.5–14.4)	0.45
platelets (k/μL) median and range	57.5, (9–553)	29.5, (7–87)	0.10
Response:			
-CR/CRi	22/29 (75.86%)	7/10 (70.00%)	0.056
-MLFS	7/29 (24.14%)	1/10 (10.00%)	
-Refractory	0 (0.00%)	2/10 (20.00%)	
MMD (EOC1)	11/29 (37.93%)	5/8 (62.50%)	0.25
MMD (EOC2)	7/29 (24.14%)	2/8 (25.00%)	>0.9999
Relapse	15 (50.00%)	7 (70.00%)	0.46
Median OS (months)	56.90	50.80	0.89
Median OS (months) (HSCT censored)	NR	NR	0.53
Median PFS (months)	62.10	14.60	0.28
Median PFS (months) (HSCT censored)	NR	24.3	0.12

All values taken at diagnosis; normal range, WBC, 4.0–11.0 k/μL; hemoglobin, 14–18 g/dL; platelets, 140–440 k/μL; Group B, with *FLT3*-ITD; Group C, with MDS-related gene mutations; BM, bone marrow; WBC, white blood cell; PB, peripheral blood; Hb, hemoglobin; CR, complete remission; CRi, CR with incomplete blood count recovery; MLFS, morphological leukemia-free status; MMD, minimal measurable disease; EOC1, end of cycle 1 induction; EOC2, end of cycle 2 induction; OS, overall survival; HSCT, hematopoietic stem cell transplantation; PFS, progression-free survival; NR, median survival not reached.

Table S5. Abnormal karyotype identified at or after relapse.

Case Number	Risk Group	Karyotype
52	B	46,XY,t(14;15)(q32;q22)[2]
58	B	46,XY,inv(1q),t(4;10),t(6;17)[3]/46,XY,t(17;20)[cp2]/46,XY,inv(1q),t(3;4)[1]/46,XY,t(1;4)[1]/46,XY [3]
61	D	46,XX,add(1)(p13),add(7)(q22),t(12;22;17)(p13;q11.2;p13)[cp3]/46,XX [8]
62	C	46,XY,del(1)(q21q22)[5]/46,Y,del(X)(q24q26)[1]/46,XY [14]

63	B	46,XX,add(2)(p11.2),del(11)(q13),add(15)(q22),der(19)t(11;19)(q13;q13.1),add(15)(q22)[1]/47,idem,+13[6]/46,XX [13]
65	B	46,XY,t(4;6)(q21;p25),add(13)(q31)[7]t(11;12)(p14;q21)[2]

Risk group assignment: Group B, with *FLT3*-ITD; Group C, with MDS-related gene mutations; Group D, with *WT1* mutation.
